# Kinetic modeling of the Calvin cycle identifies flux control and stable metabolomes in *Synechocystis* carbon fixation

**DOI:** 10.1093/jxb/ery382

**Published:** 2018-10-27

**Authors:** Markus Janasch, Johannes Asplund-Samuelsson, Ralf Steuer, Elton P Hudson

**Affiliations:** 1 Science for Life Laboratory, School of Engineering Sciences in Chemistry, Biotechnology and Health, KTH Royal Institute of Technology, Solna, Sweden; 2 Fachinstitut für Theoretische Biologie (ITB), Institut für Biologie, Humboldt-Universität zu Berlin, Berlin, Germany

**Keywords:** Calvin cycle, cyanobacteria, flux control, kinetic model, metabolic engineering, metabolic model, metabolome, parameter sampling, system stability

## Abstract

Biological fixation of atmospheric CO_2_ via the Calvin–Benson–Bassham cycle has massive ecological impact and offers potential for industrial exploitation, either by improving carbon fixation in plants and autotrophic bacteria, or by installation into new hosts. A kinetic model of the Calvin–Benson–Bassham cycle embedded in the central carbon metabolism of the cyanobacterium *Synechocystis* sp. PCC 6803 was developed to investigate its stability and underlying control mechanisms. To reduce the uncertainty associated with a single parameter set, random sampling of the steady-state metabolite concentrations and the enzyme kinetic parameters was employed, resulting in millions of parameterized models which were analyzed for flux control and stability against perturbation. Our results show that the Calvin cycle had an overall high intrinsic stability, but a high concentration of ribulose 1,5-bisphosphate was associated with unstable states. Low substrate saturation and high product saturation of enzymes involved in highly interconnected reactions correlated with increased network stability. Flux control, that is the effect that a change in one reaction rate has on the other reactions in the network, was distributed and mostly exerted by energy supply (ATP), but also by cofactor supply (NADPH). Sedoheptulose 1,7-bisphosphatase/fructose 1,6-bisphosphatase, fructose-bisphosphate aldolase, and transketolase had a weak but positive effect on overall network flux, in agreement with published observations. The identified flux control and relationships between metabolite concentrations and system stability can guide metabolic engineering. The kinetic model structure and parameterizing framework can be expanded for analysis of metabolic systems beyond the Calvin cycle.

## Teaser

An improved kinetic model of the cyanobacterial Calvin cycle employing random sampling identifies probabilistic conditions for metabolome stability and metabolic flux control, agreeing with experimental data and aiding engineering efforts.

## Introduction

The Calvin–Benson–Bassham (CBB) cycle of photosynthetic plants, algae, and cyanobacteria plays an important role in global carbon cycling (Battle, 2000), but also offers potential for industrial usage. There are ongoing efforts for enhancing Calvin cycle flux in plants to improve crop yields (Long *et al.*, 2015), but also in cyanobacteria and other autotrophs for potential use as cell factories with CO_2_ as cheap feedstock (Wijffels *et al.*, 2013; Savakis and Hellingwerf, 2015; Torella *et al.*, 2015). Recently, the Calvin cycle has been incorporated into *Escherichia coli* and other heterotrophs as a way to impart CO_2_ fixation capability (Guadalupe-Medina *et al.*, 2013; Antonovsky *et al.*, 2016; Schada von Borzyskowski *et al*., 2018). One way to improve the flux through this subnetwork is to identify the controlling catalytic steps, which then become targets for metabolic engineering. There have been several experimental efforts to identify individual limiting steps in the CBB cycle of plants (for a review, see Raines, 2003) and cyanobacteria (Liang and Lindblad, 2016), but it remains a significant challenge to provide a system-wide analysis of metabolic flux control.

Computational models of metabolism that account for rate equations and kinetic parameters of the enzymes can aid in analyzing metabolic control. Furthermore, these kinetic models provide valuable information about the dynamic behavior of the system and potential for modification (Almquist *et al.*, 2014). For example, a parameterized kinetic model can be used to estimate network stability (i.e. the ability to return to a steady state after an infinitesimal perturbation of metabolite levels). Metabolic states that are not stable could lead to lethal depletion or accumulation of metabolites upon perturbation.

Existing kinetic models of the CBB cycle are either specific for chloroplasts (Poolman *et al.*, 2000; Zhu *et al.*, 2007; Arnold and Nikoloski, 2011, Girbig *et al.*, 2012) or adaptations of these models for cyanobacteria (Jablonsky *et al.*, 2016), but all face the obstacle that kinetic models require knowledge about the parameter values for each enzymatic reaction. This information is scarce, especially for cyanobacteria, and *in vitro* kinetic parameters may not be applicable to *in vivo* conditions. Several frameworks have been developed to address this lack of information by randomly sampling the parameter space, creating thousands of parameter sets describing a specific metabolic state (defined here as metabolite concentrations and reaction rates) (Steuer *et al.*, 2006; Tran *et al.*, 2008; Mišković and Hatzimanikatis, 2011). Although these approaches differ in their details, they all can provide a probabilistic analysis of the dynamic behavior of a metabolic system at a certain steady state and thereby identify potential flux-controlling steps. While a parameter sampling approach for the CBB cycle in chloroplasts has been published, it focused on the stability of a single state and did not investigate metabolic control (Girbig *et al.*, 2012).

**Fig. 1.**
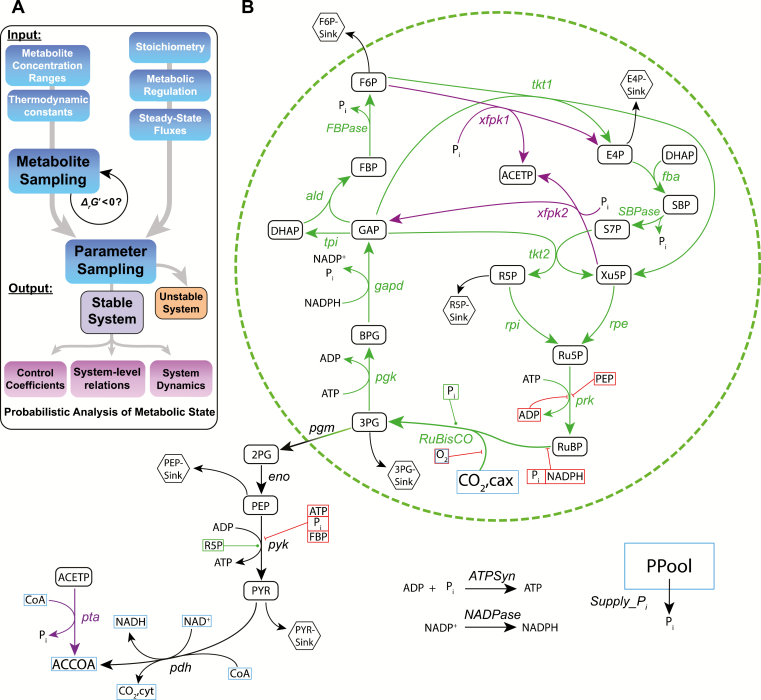
Sampling framework and model network overview. (A) Methodology for parameterizing the model structure, adapted and modified from Murabito *et al.* (2014) with addition of metabolite concentration sampling. (B) Schematic overview of all reactions and metabolites covered by the model. Reaction arrows represent the input flux directionality. Reactions in purple depict the xfpk subnetwork and reactions in black depict lower glycolysis. Red rectangles around metabolites indicate inhibitors, while green rectangles indicate activators. Hexagons represent sink metabolites and blue rectangles indicate unbalanced metabolites. 3-Phosphoglycerate (3PG), 1,3-bisphosphoglycerate (BPG), glyceraldehyde 3-phosphate (GAP), dihydroxyacetone phosphate (DHAP), fructose 1,6-bisphosphate (FBP), fructose 6-phosphate (F6P), erythrose 4-phosphate (E4P), sedoheptulose 1,7-bisphosphate (SBP), sedoheptulose 7-phosphate (S7P), xylulose 5-phosphate (Xu5P), ribose 5-phosphate (R5P), ribulose 5-phosphate (Ru5P), ribulose 1,5-bisphosphate (RuBP), 2-phosphoglycerate (2PG), phosphoenolpyruvate (PEP), pyruvate (PYR), acetyl-CoA (ACCOA), acetyl-phosphate (ACETP), inorganic phosphate (P_i_). Reactions are abbreviated as follows: Ribulose 1,5-bisphosphatase carboxylase/oxygenase (Rubisco), phosphoglycerate kinase (pgk), glyceraldehyde 3-phosphate dehydrogenase (gapd), triosephosphate isomerase (tpi), aldolase (ald), fructose 1,6-bisphosphatase (FBPase), transketolase 1/2 (tkt1/2), fructose-bisphosphate aldolase (fba), sedoheptulose 1,7-bisphosphatase (SBPase), ribulose-phosphate epimerase (rpi), phosphoribulokinase (prk), phosphoglucomutase (pgm), enolase (eno), pyruvate kinase (pyk), pyruvate dehydrogenase (pdh), phosphoketolase 1/2 (xfpk 1/2), phosphotransacetylase (pta). The abstracted cofactor supply reactions are abbreviated as ATPSyn, NADPase, and Supply_Pi.

Here, we created a kinetic model of the CBB cycle of the model cyanobacterium *Synechocystis* sp. PCC 6803 (hereafter *Synechocystis*), accounting for metabolic regulations in cyanobacteria reported in the literature as well as enzyme promiscuity. To mitigate uncertainties associated with the metabolic state due to variability in metabolomics data sets (Asplund-Samuelsson *et al.*, 2018), we initially sampled the steady-state metabolite concentrations followed by filtering based on thermodynamic feasibility. The model was subsequently parameterized around each of these metabolic states by employing random sampling of the parameter space (Fig. 1A) similar to Murabito *et al.* (2014). In contrast to recent parameter estimation and fitting frameworks (Jablonsky *et al.*, 2016), this study marks an advanced parameter sampling approach for a kinetic model of cyanobacteria, aiming for probabilistic identification of properties determining network stability and the distribution of control. The results confirm the intrinsic stability of the CBB cycle over a broad range of parameters as well as the distribution of control. Predicted trends agree with experimental findings and form the basis for further investigations that can aid metabolic engineering efforts.

## Materials and methods

### Input: model structure

A model of the central carbon metabolism of *Synechocystis* was constructed (Fig. 1B). The model comprised 29 reactions with underlying stoichiometries and kinetic rate equations, totaling 36 metabolites and 149 kinetic parameters. The model included the 13 catalytic steps of the CBB cycle, the phosphoketolase subnetwork, and reactions downstream towards acetyl-CoA (ACCOA). Branching points toward biomass were included as lumped reactions from the corresponding metabolite towards biomass sinks, based on flux balance analysis (FBA) results (see Input: computing the steady-state flux distribution). The sink reactions were implemented as irreversible Michaelis–Menten kinetics, removing the need for thermodynamic information of the products while allowing the simulation of different saturation levels. To simulate the supply of energy and redox factors in the form of ATP and NADPH, respectively, two lumped reactions representing the photosystems were included. The drain of phosphorylated metabolites via sink reactions required a phosphate supply reaction, providing free inorganic phosphate (P_i_) from an abstract summation of all phosphate sources in the cell, termed the phosphate pool (PPool). The P_i_ and PPool interconversion reaction follows mass action kinetics with equal forward and reverse rate constants. The reaction rate is therefore only dependent on the rate constant and the difference in concentration between P_i_ and PPool (for details, see Supplementary Protocol S2 at *JXB* online).

With the exception of fructose 1,6-bisphosphatase (FBPase), sedoheptulose 1,7-bisphosphatase (SBPase), the sink reactions, and the phosphate supply, the rate equations for each reaction follow reversible Michaelis–Menten-like kinetics, including equilibrium constants according to the general scheme:

$$v=\frac{V_{\text{max}}\cdot \left(\frac{A}{K_{M}^{\text{A}}}\right)\cdot \left(1-\frac{B}{A\cdot K_{\text{eq}}}\right)}{\left(1+\frac{A}{K_{M}^{\text{A}}}+\frac{B}{K_{M}^{\text{B}}}\right)}$$ (1)

for a simple conversion between two compounds *A*↔*B*. Rate equation formulations are listed in Supplementary Protocol S2.

The metabolic regulations of enzymes were taken from the literature and are included in the rate equations as competitive inhibition and/or allosteric modifiers (Supplementary Table S1). Modeling of photorespiration (oxygenase function of Rubisco) was abstracted by O_2_ acting as a competitive inhibitor for CO_2_ binding.

In the case of enzyme promiscuity, where one enzyme catalyzes two reactions, the substrates and products of the second reaction were included as competitive inhibitors for enzyme-binding sites. For example, the reactions FBPase and SBPase are catalyzed by the same enzyme. Sedoheptulose 1,7-bisphosphate (SBP) acts as an inhibitor for the FBPase reaction from fructose 1,6-bisphosphate (FBP) to fructose 6-phosphate (F6P). The value of the inhibition constant in the FBP to F6P reaction was set to be the same as the SBP binding constant *K*_M_ in the reaction of SBP to sedoheptulose 7-phosphate (S7P) (see rate equations in Supplementary Protocol S2, where the inhibition constant of SBP towards the FBPase reaction is called *K*_M_^SBP^).

### Input: defining the ‘metabolic state’

The local dynamic properties of the system, such as stability and flux control, were determined at specific metabolic (steady) states. Here, a metabolic state is defined by the sizes of the metabolite pools and the reaction rates (fluxes) connecting these pools at steady state. At steady state, the different fluxes acting on a metabolite must be balanced so that each metabolite pool stays constant over time.

### Input: computing the steady-state flux distribution

Experimental determination of reaction fluxes in *Synechocystis* is scarce due to the challenging traceability of autotrophic metabolism (Young *et al.*, 2011; Adebiyi *et al.*, 2015; Hendry *et al.*, 2017, Gopalakrishnan *et al.*, 2018). We therefore computed a flux distribution at steady state using FBA on a genome-scale model of *Synechocystis* (Knoop *et al.*, 2013) during autotrophic growth [COBRA-toolbox (Schellenberger *et al.*, 2011) in MATLAB v. R2015a]. A detailed description of the flux simulation is provided in Supplementary Protocol S3, and the resulting flux distribution used as input for the parameter-sampling algorithm is listed in Supplementary Table S2.

### Input: generation of feasible metabolomes using random sampling

In addition to the fluxes, the metabolic state of the system is also defined by the metabolite concentrations. Published experimental metabolomics data sets for *Synechocystis* show high variability under similar cultivation conditions (Asplund-Samuelsson *et al.*, 2018).

By employing network-embedded thermodynamic analysis (Kümmel *et al.*, 2006; Zamboni *et al.*, 2008), Asplund-Samuelsson *et al.* (2018) identified metabolite concentration ranges in which biomass formation in *Synechocystis* was thermodynamically feasible.

To combat the uncertainties associated with the published data sets, a random sampling approach was used to cover the whole, thermodynamically allowable metabolite concentration space. In short, for each metabolite, a random value within the concentration ranges was sampled in log space, resulting in sets of metabolite concentrations spanning the concentration space evenly. The concentration ranges for each metabolite are listed in Supplementary Table S3. The resulting sets were tested for their thermodynamic feasibility by calculating the change in Gibbs free energy of each reaction (see also Supplementary Protocol S3). Concentration sets not providing every reaction with a negative change in Gibbs free energy in their pre-defined directions (i.e. thermodynamically infeasible) were discarded. To ensure biologically reasonable metabolite concentrations, their total sum could not exceed 100 mM. This limit reflects osmotic constraints as well as experimental observations that the total metabolite concentration is ~200 mM across several cell types (Park *et al.*, 2016), while it also reserves 100 mM for non-CBB metabolites not included in the model. The size of the PPool was sampled as a multiple of the P_i_ concentration, ranging between 1.1× and 5×. Cofactor concentrations were sampled with the additional constraint of growth-compatible and thermodynamically feasible cofactor pair ratios (ATP/ADP, NADP/NADPH, and NAD/NADH) (Asplund-Samuelsson *et al.*, 2018, see Supplementary Protocol S3 for details). All feasible metabolite concentration sets (fMCSs) are listed in Supplementary Table S4. The metabolite sampling was performed in MATLAB v. R2015b on an Ubuntu 16.04 Linux system using 16 cores at 2.4 GHz, taking up to 75 min.

### Output: sampling of kinetic parameters and testing of system stability

To determine the local dynamic behavior of a system at a certain metabolic state, namely its stability and response to perturbation, the kinetic parameters of the enzymes must be known. The combination of metabolite concentrations and kinetic parameters also defines the saturation level of the enzymes at this steady state.

As there is a general lack of available parameter values and uncertainty in the validity of *in vitro* parameters *in vivo*, we used a random sampling approach to estimate enzyme parameters for each fMCS, similar to Murabito *et al.* (2014).

Specifically, for each Michaelis–Menten, inhibition, and activation constant (*K*_M_, *K*_i_, and *K*_a_, respectively) in the model, a value was randomly picked from a range of 0.01× to 100× around the corresponding metabolite concentration (translating to 1–99% enzyme saturation).

The sampling was performed in the log space, ensuring an even coverage of the whole parameter space.

In essence, the algorithm determined the saturation state of the enzyme for the corresponding metabolites, according to

$$v=V_{\text{max}}\times f\left(X,K_{\text{M,i,a}},K_{\text{eq}}\right)$$ (2)

The *V*_max_ values for each reaction were then determined by matching the rate equation containing the previously sampled parameters and concentrations (function *f*) with the steady-state input flux *v*^0^ of the corresponding reaction, via

$$V_{\text{max}}=v^{0}/f\left(X,K_{\text{M,i,a}},K_{\text{eq}}\right)$$ (3)

This process was repeated 1000 times for each of the metabolite concentration sets, leading to sets of kinetic parameters describing a possible metabolic state of the system that covered most of the allowable parameter space.

The stability of each combination of sampled sets was analyzed mathematically by determining the eigenvalues of the reduced Jacobian matrix at the metabolic state (*X*^0^), where a negative real-part for all eigenvalues indicated a stable steady state:

$$J^{a}=S^{\text{a}}\cdot {\frac{\partial v}{\partial X}|}_{X^{\text{0}}}\cdot L$$ (4)

where *J'* represents the reduced Jacobian matrix. *S'* is the reduced stoichiometric matrix having full rank, and *L* is the link matrix with *S*=*L*×*S'*; see Reder (1988) for a detailed description.

Note that in this context stability refers to the ability of the system to return to its initial state after an infinitesimal perturbation of the state variables (i.e. the metabolite concentrations).

The parameter sampling was performed in MATLAB v. R2015b on an Ubuntu 16.04 Linux system using 16 cores at 2.4 GHz, taking ~1 h.

### Output: determining flux control coefficients (FCCs)

Parameterizing the model at a certain stable metabolic state allowed determination of control coefficients (Kacser and Burns, 1995) for the parameter sets, meaning that for each fMCS, the control coefficients for all of the corresponding stable parameter sets of this fMCS were determined. The coefficients were calculated by linear analysis at the steady state via the Jacobian, simulating infinitesimal perturbations in activity, as described (Reder 1988; Murabito *et al.*, 2014), with the concentration control coefficients defined as:

$$C^{X}=-D_{X^{0}}^{-1}\times L\times J^{a}{}^{-1}\times S^{a}\times D_{v^{0}}$$ (5)

which in turn were used to calculate the flux control coefficients via

$$C^{J}=1+D_{v^{0}}^{-1}\times {\frac{\partial v}{\partial X}|}_{X^{0}}\times D_{X^{0}}\times C^{X}$$ (6)

with $D_{X^{0}}$ and $D_{v^{0}}$ denoting diagonal matrices for metabolite concentrations and fluxes, respectively (Reder 1988).

### Output: clustering of FCC patterns

Some reactions may be similar in how they exert control over other reactions. We performed a hierarchical clustering analysis in R v. 3.4.3 in order to investigate the similarity of FCC patterns of effector reactions (exerting control, i.e. reactions whose rate is perturbed) as well as target reactions (experiencing control, i.e. reactions whose flux is affected by the perturbation in the effector reaction rate). To prepare the data for analysis, the FCCs were rounded to zero if the absolute value was <1 × 10^–6^ and then inverse hyperbolic sine transformed (function asinh). A tenth of the effector and target FCC patterns, selected by random sampling (sample), were used in the subsequent steps due to limited computational capacity. To reduce the complexity of the data set further and extract the most prominent patterns, which, due to covariance, might not be reflected in the FCC medians, principal component analysis (PCA) was conducted. The data were scaled (scale), reduced to patterns with finite values, subjected to PCA (prcomp), and rotated to the PCs (*x* variable of the prcomp output), yielding 29 by 29 matrices representing the patterns of control exertion or experience for the 29 reactions of the model. The matrices containing a reduced representation of the full effector and target pattern data sets were hierarchically clustered with multiscale bootstrap resampling (10 000 bootstraps) using the pvclust function (Suzuki and Shimodaira, 2006) at default settings. The clustering was visualized as dendrograms, with approximately unbiased *P*-values indicating significantly similar FCC patterns.

All files, input data sets, and scripts used in this study are found in Supplementary Protocol S1 and can be accessed at https://github.com/MJanasch/CBB_Kinetics, allowing for recreating every sampling and analysis step [e.g. the >3 million parameter sets generated here (~28 GB)]. The sampling processes in MATLAB were parallelized using GNU Parallel v. 20141022 (Tange, 2011).

## Results and Discussion

The kinetic model of the CBB cycle developed here was used to investigate factors influencing stability (i.e. the ability of the system to return to a steady state after an infinitesimal perturbation of the metabolite concentrations) and distribution of flux control (i.e. how network reactions, when their rates are altered, affect the rates of other network reactions). The reactions and metabolites covered by the model are depicted in Fig. 1B. The model included enzyme promiscuity for reactions catalyzed by the same protein as well as metabolic regulations reported for cyanobacteria, which have been found to differ from those in chloroplasts (Tamoi *et al.*, 1998; Yan and Xu, 2008). The phosphoketolase (xfpk) reactions are not found in higher plants and provide a shortcut from CBB cycle intermediates towards ACCOA, thus playing a major role in the central carbon metabolism in *Synechocystis* (Xiong *et al.*, 2015) as well as engineered pathways (Anfelt *et al.*, 2015).

The workflow for determining enzyme kinetic parameters in the CBB model is shown in Fig. 1A and described in the Materials and methods. Ideally, a known metabolome and flux distribution is used as a basis for parameterization. However, experimental data sets in *Synechocystis* are associated with a large uncertainty (Asplund-Samuelsson *et al.*, 2018). To avoid relying on a single or a small number of metabolome data sets and their uncertainty, sets of metabolite concentrations that are thermodynamically feasible were generated by random sampling (feasible metabolite concentration sets; fMCSs). Specifically, 5 × 10^7^ sampling rounds resulted in 3135 fMCSs.

Then, random sampling of kinetic parameters (containing *V*_max_ and *K*_M_ for all enzymes) was employed to obtain 1000 parameter sets for each fMCS, with the constraint that computed flux distributions for each parameter set match those obtained by FBA.

Lastly, the stability of each parameter set was checked using linear analysis of the Jacobian (Materials and methods), and FCCs were determined.

While we focus our analysis on metabolome stability, enzyme saturation, and flux control, we emphasize that by providing the data sets and scripts to recreate every step, the model and the data sets can be further analyzed by the scientific community.

### The Calvin cycle is stable across many metabolic states

Identifying conditions enabling a stable steady state is crucial for engineering modifications of the system. The stability conditions are also indicators of possible real metabolic states of the system, as an unstable steady state would not be sustained in nature due to accumulation or depletion of essential metabolites upon perturbation (Theisen *et al.*, 2016). Constant environmental fluctuations in nature or bioreactors render unstable states infeasible and the system moves to a different, possibly stable, state. This provides further guidance in metabolic engineering, as modifications moving the metabolism into a state of lower stability could cause non-viable cells or low productivity (Theisen *et al.*, 2016).

A large fraction of the 1000 random parameter sets generated for each fMCS were stable, ranging from 24.6% to 92.9% stable parameter sets, with a median of 66.8%.

The high number of stable steady states, despite the broad sampling range for metabolite concentrations and enzyme parameters, showed the intrinsic stability of the Calvin cycle network structure, which was also found in prior studies for chloroplasts (Girbig *et al.*, 2012). All fMCSs and their fraction of parameter sets resulting in stable steady states are listed in Supplementary Table S4.

The broad sampling of metabolite concentrations included values at the edge of or beyond measured physiological values and thus enabled the exploration of the whole thermodynamically feasible space. Sampling these metabolite ranges is of special interest for metabolic engineering, where the metabolism is modified beyond the wild-type ranges.

**Fig. 2.**
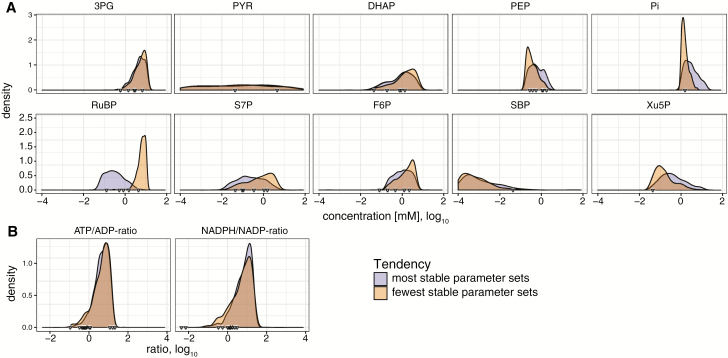
Tendencies of selected sampled metabolite concentrations (A) and ratios (B) towards stability. Density of metabolite concentrations in the top (purple; most stable parameter sets) and bottom (orange; fewest stable parameter sets) deciles (10%) of fMCSs according to the number of stable steady states. Concentration (in mM) and ratio are presented as log_10_ values on the *x*-axis. Triangles on the *x*-axis indicate published values used for determining concentration ranges in Asplund-Samuelsson *et al.* (2018).

Figure 2A shows the concentration ranges covered by fMCSs for selected metabolites constrained by input ranges and thermodynamics (for all metabolites, see Supplementary Figs S1 and S2). Some metabolites were constrained to high values (3-phosphoglycerate), while other spanned a wide range [pyruvate (PYR)]. The ATP/ADP and NADPH/NADP ratios, displayed in Fig. 2B, were accumulated in the high end of their range. We suspect that this resulted from ATP and NADPH mainly being used as substrates, favoring a high concentration, and were provided via two lumped reactions with favorable thermodynamics, representing the photosystems.

### High concentrations of RuBP are associated with system instability

The sampling of fMCSs and kinetic parameter sets allowed for a probabilistic analysis of the relationship between a metabolite concentration and stability. Figure 2A shows the propensities of certain metabolite concentrations to correlate with the number of stable systems (for all metabolites, see Supplementary Fig. S2). Most metabolites showed no distinct tendency towards favoring more or fewer stable steady states. However, a distinguishable enrichment of stable steady states was evident for low concentrations of ribulose 1,5-bisphosphate (RuBP), S7P, and F6P. Conversely, there were fewer stable states associated with low concentrations of phosphoenolpyruvate (PEP), xylose 5-phosphate (Xu5P), SBP, and P_i_. Of all of these metabolites, only RuBP showed no accumulation bias due to the thermodynamic constraints on the fMCSs (Supplementary Fig. S1).

Additionally, Fig. 2 (and see Supplementary Fig. S2) includes data points for published metabolomics data used to determine the concentration ranges in Asplund-Samuelsson *et al.* (2018). Most of these data points lie in the areas of high stability, which supports the validity of our analysis.

The main function of the CBB cycle is to recycle RuBP for another round of CO_2_ fixation via the carboxylation function of Rubisco, thus giving RuBP a central role in the network. The association of high RuBP concentrations with instability in the Calvin cycle has direct implications for metabolic engineering efforts, as modifications that increase the amount of RuBP, such as overexpression of phosphoribulokinase (prk) or repression of Rubisco, may render the metabolic network less robust. Similar conclusions could be drawn for all distinctive patterns, such as for PEP, Xu5P, and SBP mentioned above.

Interestingly, RuBP ‘toxicity’ has been reported for *Escherichia coli* (Parikh *et al.*, 2006) and *Rhodospirillum rubrum* (Wang *et al.*, 2011), where it was mostly associated with an impaired balance between prk and Rubisco. RuBP toxicity was also suggested in recent failed experimental attempts to overexpress the *prk* gene in cyanobacteria (Kanno *et al.*, 2017). Expression of the *prk* gene without that for Rubisco impaired growth in *E. coli* (Hudson *et al.*, 1992*a*), as RuBP accumulates as a ‘dead-end’ metabolite.

The tendency of high RuBP to lead to reduced stability in the CBB cycle could hint at the importance of the balance between the activities of prk and Rubisco to be able to react to perturbations. Notably, no wild-type value for RuBP has been published for values exclusively in the unstable area (Fig. 2), and values from mutants might be difficult to obtain due to lower viability. Further experimental investigations could provide insight into the exact mechanisms of the reported RuBP toxicity and its association with enzyme balance.

### Low substrate saturation and high product saturation of promiscuous enzymes favor stability

The sampling of fMCS and kinetic parameter sets also allowed for a probabilistic analysis of the relationship between stability and enzyme saturation levels, namely [S]/*K*_M_ for either the forward or reverse reaction. Following Michaelis–Menten-style rate laws, a low enzyme saturation results in high sensitivity of the reaction rate to the specific metabolite, while changes in metabolite concentration at high enzyme saturation have less influence. An enzyme with low substrate saturation and high product saturation can thus buffer changing substrate concentrations since excess metabolite can be used up via an increased reaction rate. With high product saturation, product pool changes caused by changes in reaction rate will not counteract the buffering effect strongly.

**Fig. 3.**
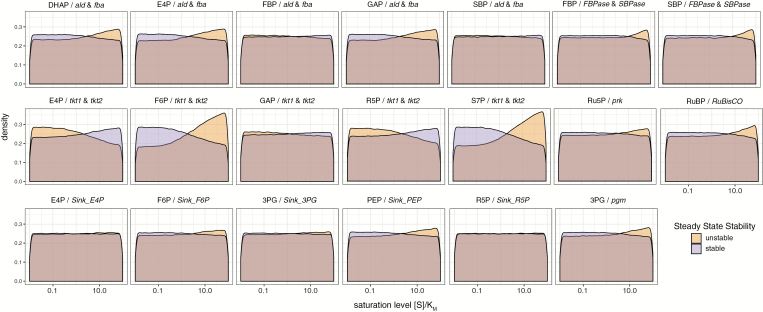
Tendencies of saturation states towards stability and instability for selected enzymes. Density of selected enzyme saturation levels, defined as the metabolite concentration divided by the associated *K*_M_ value, depicted on a log_10_ scale. Purple and orange refer to stable and unstable states, respectively. The header for each histogram states the metabolite–reaction(s) pair to which the saturation refers.

Most enzymes showed equal substrate and product saturation levels for stable and unstable states, smoothly distributed across the entire possible range (saturation 1–99%), again emphasizing the overall high predisposition for stability of the CBB cycle (Fig. 3; for all enzymes, see Supplementary Fig. S3). However, several enzymes had reactions where low substrate saturation and high product saturation were associated with stability, including the promiscuous enzymes aldolase (ald) and fructose-bisphosphate aldolase (fba), FBPase and SBPase, and transketolase 1 (tkt1) and transketolase 2 (tkt2). The xfpk reactions were an exception to this trend. Note that FBPase and SBPase do not have product saturation since they were modeled as irreversible. The reaction pairs catalyzed by these enzymes form a highly interconnected part of the CBB cycle responsible for RuBP regeneration (upper right in Fig. 1B). Each metabolite influenced more than one of the involved enzymatic rate equations via the modeled promiscuity; the product of one reaction either served as the substrate for another, or acted as competitive inhibitors in other reactions. Based on the saturation trends described above, a stable state was promoted by a consecutive buffering effect through the network, reaching prk and Rubisco.

Most trends in Fig. 3 can be exemplified by the saturation of ald by its substrates dihydroxyacetone phosphate (DHAP) and glyceraldehyde 3-phosphate (GAP). Increased stability was observed when saturation was between 1% and 50%, the latter corresponding to [S]=*K*_M_. Saturation >50% was associated with lower stability. This case could mark a compromise in the trade-off between flux sensitivity and efficient enzyme usage, as low saturation would necessitate high enzyme abundance to enable the flux. This high protein burden is associated with metabolic costs and poses possible molecular crowding issues (Beg *et al.*, 2007; Bennett *et al.*, 2009).

Another hypothesis is that metabolite concentrations in central carbon metabolism have to be close to their associated *K*_M_ values because some reactions must switch directions depending on carbon source. This hypothesis is supported by systemic comparisons of metabolomics data and published *K*_M_ values in *E. coli* (Bennett *et al.*, 2009) and other heterotrophic organisms (Park *et al.*, 2016). Our data suggest a similar case can be observed in cyanobacteria. Dark conditions require breakdown of storage compounds mainly via glycolysis and the oxidative pentose-phosphate pathway, which employ to a large extent the same enzymes used in the Calvin cycle, but in the opposite direction (Yang *et al.*, 2002; Knowles and Plaxton 2003).

All sink reactions in the model (excluding R5P and PYR) followed the same tendency of low substrate saturation associated with stability (Fig. 3). Low saturation for the sink reactions agrees with the conclusions of a recent theoretical study of the stability of autocatalytic cycles (Barenholz *et al.*, 2017). That study concluded that at least one of the reactions branching off from an autocatalytic cycle (such as the CBB) must have a lower saturation, regarding the mutual substrate, compared with the autocatalytic reaction that continues the cycle. Furthermore, an experimental study showed that stable operation of the Calvin cycle in *E. coli* could only be reached after laboratory evolution changed the sink reaction for R5P so that the catalyzing enzyme had a lower saturation level (Antonovsky *et al.*, 2016). This also explains why there was no tendency towards stability for the sink reaction acting on PYR, as it is outside any autocatalytic cycle covered by our model. The absence of influence of the R5P sink could be explained by the high number of sink reactions and only one has to follow the trend identified in Barenholz *et al.* (2017). Note that phosphoglucomutase (pgm) acts as a branching reaction from the CBB cycle and also follows the trend.

There are several reports of genetic instability in metabolically engineered cyanobacteria (Jones, 2014), where production cells revert to wild type through mutation of the heterologous pathway. This instability has been interpreted as cells escaping the burden of product toxicity, metabolite imbalance, or forced carbon flux away from biomass formation (Du *et al.*, 2017), but metabolic instability as described here has not been considered. The kinetic model developed here could be used to interpret these systems, as a combination of metabolite draining by the production pathway and cultivation conditions could cause production strains to enter an unstable metabolic state.

### Metabolic control in the Calvin cycle is distributed among several enzymes

**Fig. 4.**
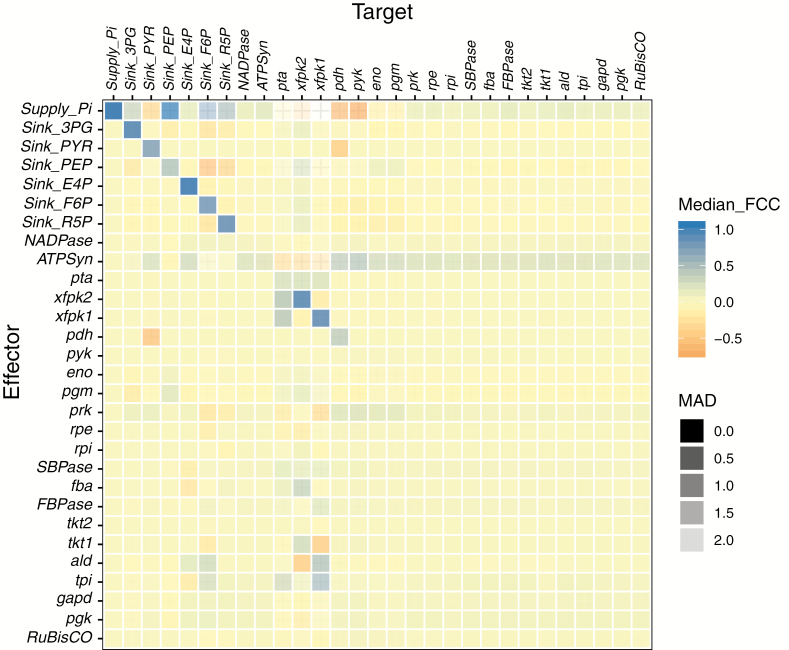
Median FCC values from all stable parameter sets for all fMCSs, depicting how an infinitesimal change in effector rate affects the flux through the target reaction. Blue corresponds to a positive change and red to a negative change in flux. Higher transparency corresponds to a larger median absolute deviation (MAD) of the distribution of FCCs from which the median is calculated.

Engineering improvements to the Calvin cycle is aided by knowledge of which enzymes control flux. An FCC is a quantitative measure of how a change in the rate of a selected reaction (‘effector reaction’) affects the flux through any reaction in the system (‘target reaction’). Positive or negative FCCs refer to an increase or decrease of the flux following a change in enzyme activity, respectively. A linear approximation method based on infinitesimal changes in activity (not probing activity changes of large magnitudes) was used to determine the FCCs for each stable parameter set. This approach resulted in distributions of FCCs for each enzyme and target reaction pair (median FCC and median absolute derivation; Fig. 4). Inspection of these FCC distributions revealed that most median FCCs were significantly lower than 1 (i.e. no one reaction is the ‘bottleneck’ of the Calvin cycle). A distributed flux control is expected from highly interconnected and complex metabolic networks (Stephanopoulos *et al.*, 1998).

However, some reactions offered positive control over many other reactions, meaning an increase in the rate of the former corresponded to an increase in flux in the latter. The most prominent effector reactions were ATP synthase (ATPSyn), prk, phosphoglycerate kinase (pgk), SBPase, and FBPase. Weaker effector reactions were glyceraldehyde 3-phosphate dehydrogenase (gapd), triosephosphate isomerase (tpi), tkt1 and fba.

The positive control of ATPSyn emphasized the direct dependence of the CBB cycle on reactions of the photosystems, which generate ATP and NADPH. Similarly, the positive control of Supply_P_i_ underlined the dependency on free inorganic phosphate supply.

The predicted positive effects of increasing the activity of SBPase/FBPase, fba, and tkt1 correlated well with recent experiments overexpressing these enzymes in *Synechocystis* (Liang and Lindblad, 2016) and *Synechococcus* sp. 7002 (De Porcellinis *et al.*, 2018), and are further supported by findings in plants (Raines, 2003; Miyagawa *et al.*, 2001). The artificial phosphate supply reaction, Supply_P_i_, showed positive control over reactions in the CBB cycle and negative control over reactions in the downstream pathway to ACCOA, probably caused by the inhibition of pyruvate kinase by P_i_.

Rubisco showed small but positive control over many reactions. This enzyme has received much attention in efforts to improve the CBB cycle (Whitney *et al.*, 2011; Ducat and Silver, 2012), as it catalyzes the actual carbon fixation reaction. Experimental studies of overexpression of Rubisco in cyanobacteria are not conclusive regarding its effect on growth rate (Marcus *et al.*, 2011; Liang and Lindblad, 2017; De Porcellinis *et al.*, 2018), and several experimental investigations of Rubisco activity in plants showed only limited influence under a wide range of conditions (Quick *et al.*, 1991; Hudson *et al*., 1992*b*; Raines, 2003). Taken together, these findings support the prediction that Rubisco is not a major controlling element in the CBB cycle, at least under the conditions covered by the model.

Contrary to our results, prk was reported to have no flux control in tobacco (Paul *et al.*, 1995), as an effect on growth was only detected when activity was reduced by >85%. pgk, gapd, and tpi (gluconeogenesis) exerted positive control only over the reactions directly in the CBB cycle, and showed only a small effect on the downstream reactions towards ACCOA. This could be because the reactions constitute the first steps in recycling carbon back into the core network finally to restore RuBP, enabling another round of fixing a molecule of CO_2_.

### Network structure gives rise to group control and non-trivial relationships

Control can be exerted over parts of the metabolism by reactions that seem to have no direct connection. One example of such network effects is prk, which exerted negative control over the sinks for R5P and F6P, which are relatively close to prk, but also positive control over the downstream pathway to ACCOA, relatively far away in the network. The most probable explanation was a sequential effect, where the reactions in a linear pathway influence each other one after another. Notably, Rubisco and pgk were not influenced as strongly by this effect. The effect on the sink reaction for F6P might result from the connection via P_i_ through ATPSyn. These non-trivial observations mark targets for further research, for example through experimental investigations and modeling in a larger metabolic context where the abstracted sink reaction is formulated in more detail.

The patterns of FCCs discernible in Fig. 4 indicate that some reactions exert or experience control in a similar fashion. To confirm that this is the case, and also for a wider scope than just the medians, we applied PCA and clustered the reactions according to their patterns of exerting control (as effectors) and being controlled (as targets) (Materials and methods; Supplementary Fig. S4, S5). The clustering analysis revealed that the main reactions of the CBB cycle are controlled in a coupled manner; their fluxes tend to be affected together (Supplementary Fig. S5). The high connectivity between the reactions in these groups may explain the coupled control. A separate clustering was observed for downstream reactions towards ACCOA, the sink reactions, and the xfpk subnetwork. The clustering of reactions according to their ability to exert control (Supplementary Fig. S4) provides further insight for metabolic engineers to choose between different targets to cause similar effects upon perturbation.

### The phosphoketolase pathway exerts only negligible control over other reactions

The xfpk reactions and phosphotransacetylase (pta) exerted virtually no control over any other reaction but themselves. This emphasizes the parallel character of the subnetwork formed by the xfpk reactions towards ACCOA (Supplementary Fig. S4), bypassing the traditional Calvin cycle and downstream reactions that include decarboxylation of PYR (Bogorad *et al.*, 2013; Xiong *et al.*, 2015; Anfelt *et al.*, 2015). This shortcut has been suggested to assist energy balancing during heterotrophic growth, but also to support carbon channeling into the tricarboxylic acid (TCA) cycle under photosynthetic conditions (Xiong *et al.*, 2015).

The three reactions in the xfpk subnetwork were, however, affected by many reactions. The negative control of tkt1 over xfpk1 could result from a competition for the same substrate (i.e. F6P), whereas the positive control on xfpk2 could result from the negative effect on xfpk1 combined with the supply of substrate for xfpk2 (i.e. Xu5P). Reasons for the reversed control of ald on xfpk1 and xfpk2 were not as obvious as simple substrate competition, and the relationships between the reactions were probably caused by the aforementioned non-trivial network effects in combination with the enzyme promiscuity interconnecting the two reactions, as they are catalyzed by the same enzyme. Further investigations will be necessary to understand this relationship fully.

### How can we increase the predictive capability of kinetic models?

The model reproduced known and reasonable behavior and predicted trends that are in good agreement with published experimental data, displaying the strength of the random sampling approach and the subsequent probabilistic analysis. The capability to explore the whole metabolic and parameter space without constraining the search to finding a single data set fitted to the input data expands the potential of the obtained results and avoids uncertainty-related overfitting issues.

To improve the predictive strength of this framework, advanced omics technologies can be employed to constrain further the broad sampling boundaries employed here to capture the physiological metabolic state more accurately.

In addition to the previously discussed benefits of more targeted metabolomics studies (Asplund-Samuelsson *et al.*, 2018), fluxomics data sets from different conditions and mutants would aid the understanding of the system in substantial ways; this is an ongoing endeavor (Gopalakrishnan *et al.*, 2018) and especially challenging for autotrophic metabolism (Adebiyi *et al.*, 2015). Additionally, the analysis of quantitative proteomic data sets would reveal the abundance ratios of enzymes, enabling more accurate modeling of *in vivo* fluxes and the role of enzyme abundance in the network context (Reimers *et al.*, 2017; Sánchez *et al.*, 2017).

Computational studies by Jablonsky *et al.* (2013, 2014, 2016) have identified the parallel expression of isoenzymes with different kinetic properties as important influencers for metabolic regulation in the Calvin cycle. The results from our simplified analysis could therefore be refined by incorporating ratios between particular isoenzymes, sampling their specific kinetics individually, and investigating their influence on stability of metabolic states. The possibility of redundancy caused by several isoenzymes catalyzing the same reaction is furthermore of special interest when trying to modify the flux through a certain reaction.

While the model is formulated with kinetic rate equations describing the enzymatic reactions, to the best of our knowledge, complex reaction mechanisms and possible allosteric and competitive effectors might not yet have been discovered. By randomly assigning different types of regulators, the modeling approach used in this study could provide guidance in finding possible regulatory mechanisms influencing state stability. Furthermore, the activities of several enzymes of the Calvin cycle are regulated by protein interaction via thioredoxin (Raines, 2003) or CP12 (Tamoi *et al.*, 2005), which play important roles in investigating the dynamics between light and dark conditions as well as engineering efforts.

Finally, expanding the model to cover larger parts could further increase the predictive strength, since it would reveal more complex dynamics between the parts of the highly interconnected central carbon metabolism.

### Conclusions

Kinetic relationships are crucial in uncovering controlling steps in core metabolic networks such as the Calvin cycle. This study aids to reduce the uncertainty related to possible metabolic states of the Calvin cycle by employing a kinetic model and random sampling, which allowed investigation of dynamic properties such as stability and the distribution of control. Stability correlated with low abundances of the metabolite RuBP, suggesting that accumulation of this metabolite is not tenable. Furthermore, saturation levels of some promiscuous enzymes were also associated with instability. In general, flux control over the Calvin cycle is distributed over several reactions. The results of this study can guide further investigations into the dynamics of the Calvin cycle as well as its modifications by metabolic engineering for improved performance or transplantation into other hosts. The combination of thermodynamic feasibility and steady-state stability presented here constrains the solution space of metabolic models towards a more realistic description of reaction networks in nature.

## Supplementary data

Supplementary data are available at *JXB* online.

Protocol S1. Model structure file and scripts for reproducing the sampling and the plots.

Protocol S2. List of rate equations for all reactions in the model.

Protocol S3. Detailed description of flux balance analysis conditions for calculating the input fluxes and thermodynamic analysis of the sampled metabolite concentration sets.

Table S1. Metabolic regulators of the rate equations.

Table S2. Input fluxes for every reaction used in the parameter sampling, derived from flux balance analysis.

Table S3. Input ranges for metabolite concentration sampling, with minimum and maximum values for each metabolite.

Table S4. All feasible metabolite concentration sets resulting from random metabolite sampling and their associated percentage stability from the parameter sampling.

Fig. S1. Distribution of sampled metabolite concentrations for all thermodynamically feasible sets.

Fig. S2. Tendencies of all metabolite concentrations towards more or fewer stable steady states based on the top and bottom deciles of all fMCSs for 1000 parameter sampling rounds.

Fig. S3. Tendencies of saturation states of all enzymes towards association with stability and instability in the system.

Fig. S4. Clustering of reactions by their FCCs according to their property of exerting control.

Fig. S5. Clustering of reactions by their FCCs according to their property of being controlled.

Supplementary Table

Supplements Protocols Figures

Supplementary Material

## Abbreviations:

ACCOA: acetyl-CoA
ald: aldolase
ATPSyn: ATP synthase
CBB: Calvin–Benson–Bassham
fba: fructose-bisphosphate aldolase
FBA: flux balance analysis
FBP: fructose 1,6-bisphosphate
FBPase: fructose 1,6-bisphosphatase
FCC: flux control coefficient
fMCS: feasible metabolite concentration set
F6P: fructose 6-phosphate
gapd: glyceraldehyde 3-phosphate dehydrogenase
PEP: phosphoenolpyruvate
pgk: phosphoglycerate kinase
Pi: inorganic phosphate
PPool: phosphate pool
prk: phosphoribulokinase
PYR: pyruvate
R5P: ribose 5-phosphate
RuBP: ribulose 1,5-bisphosphate
Ru5P: ribulose 5-phosphate
S7P: sedoheptulose 7-phosphate
SBP: sedoheptulose 1,7-bisphosphate
SBPase: sedoheptulose 1,7-bisphosphatase
tkt1/2: transketolase 1/2
tpi: triosephosphate isomerase
xfpk1/2: phosphoketolase 1/2
Xu5P: xylulose 5-phosphate
