# Supplementary material for: Kinetic modeling of the Calvin cycle identifies flux control and stable metabolomes in Synechocystis carbon fixation
Source: J Exp Bot. 2018 Oct 27;70(3):973–83. doi: 10.1093/jxb/ery382 (PMC6363089; doi:10.1093/jxb/ery382)
Supplement: Supplements Protocols Figures [file ery382_suppl_supplements_protocols_figures.pdf]

Supplementary Protocol S2. List of rate equations for all reactions in the model.

RuBisCO

$$\frac{\left(\frac{P_i}{P_i+Ka^{P_i}}\right)*Vmax*\left(\frac{RuBP*CO_{2}\_cax}{CO_{2}\_cax+Km^{CO_{2}\_cax}*(1+\frac{O_2}{Km^{O_2}})}\right)}{RuBP*Km^{RuBP}*\left(1+\frac{P_i}{Ki^{P_i}}+\frac{NADPH}{Ki^{NADPH}}\right)}$$

pgk

$$\frac{Vmax*\left(\frac{P3P*ATP}{Km^{P3G}*Km^{ATP}}\right)*\left(1-\frac{BPG*ADP}{P3G*ATP*K_{eq}}\right)}{\left(1+\frac{P3G}{Km^{P3G}}+\frac{BPG}{Km^{BPG}}\right)*\left(1+\frac{ATP}{Km^{ATP}}+\frac{ADP}{Km^{ADP}}\right)}$$

gapd

$$\frac{Vmax*\left(\frac{BPG*NADPH}{Km^{BPG}*Km^{NADPH}}\right)*\left(1-\frac{GAP*NADP*P_i}{BPG*NADPH*K_{eq}}\right)}{\left(1+\frac{NADP}{Km^{NADP}}+\frac{NADPH}{Km^{NADPH}}\right)*\left(1+\frac{GAP}{Km^{GAP}}+\frac{BPG}{Km^{BPG}}+\frac{P_i}{Km^{P_i}}\right)}$$

tpi

$$\frac{Vmax*\left(\frac{GAP}{Km^{GAP}}\right)*\left(1-\frac{DHAP}{GAP*K_{eq}}\right)}{\left(1+\frac{GAP}{Km^{GAP}}+\frac{DHAP}{Km^{DHAP}}\right)}$$

ald

$$\frac{Vmax*\left(\frac{DHAP*GAP}{Km^{DHAP}*Km^{GAP}}\right)*\left(1-\frac{FBP}{DHAP*GAP*K_{eq}}\right)}{\left(1+\frac{FBP}{Km^{FBP}}\right)*\left(1+\frac{SBP}{Km^{SBP}}\right)+\left(1+\frac{DHAP}{Km^{DHAP}}\right)*\left(1+\frac{E4P}{Km^{E4P}}\right)*\left(1+\frac{GAP}{Km^{GAP}}\right)-1}$$

*tkt*1

$$\frac{Vmax*\left(\frac{F6P*GAP}{Km^{F6P}*Km^{GAP}}\right)*\left(1-\frac{E4P*Xu5P}{F6P*GAP*K_{eq}}\right)}{\left(1+\frac{F6P}{Km^{F6P}}+\frac{E4P}{Km^{E4P}}\right)*\left(1+\frac{GAP}{Km^{GAP}}+\frac{Xu5P}{Km^{Xu5P}}\right)*\left(1+\frac{S7P}{Km^{S7P}}+\frac{R5P}{Km^{R5P}}\right)}$$

*tkt*2

$$\frac{Vmax*\left(\frac{S7P*GAP}{Km^{S7P}*Km^{GAP}}\right)*\left(1-\frac{R5P*Xu5P}{S7P*GAP*K_{eq}}\right)}{\left(1+\frac{F6P}{Km^{F6P}}+\frac{E4P}{Km^{E4P}}\right)*\left(1+\frac{GAP}{Km^{GAP}}+\frac{Xu5P}{Km^{Xu5P}}\right)*\left(1+\frac{S7P}{Km^{S7P}}+\frac{R5P}{Km^{R5P}}\right)}$$

FBPase

$$\frac{Vmax*FBP}{FBP+Km^{FBP}*\left(1+\frac{SBP}{Km^{SBP}}\right)}$$

fba

$$\frac{Vmax*\left(\frac{DHAP*E4P}{Km^{DHAP}*Km^{E4P}}\right)*\left(1-\frac{SBP}{DHAP*E4P*K_{eq}}\right)}{\left(1+\frac{FBP}{Km^{FBP}}\right)*\left(1+\frac{SBP}{Km^{SBP}}\right)+\left(1+\frac{DHAP}{Km^{DHAP}}\right)*\left(1+\frac{E4P}{Km^{E4P}}\right)*\left(1+\frac{GAP}{Km^{GAP}}\right)-1}$$

SBPase

$$\frac{Vmax*SBP}{SBP+Km^{SBP}*\left(1+\frac{FBP}{Km^{FBP}}\right)}$$

rpi

$$\frac{Vmax*\left(\frac{R5P}{Km^{R5P}}\right)*\left(1-\frac{Ru5P}{R5P*K_{eq}}\right)}{\left(1+\frac{Ru5P}{Km^{Ru5P}}+\frac{R5P}{Km^{R5P}}\right)}$$

rpe

$$\frac{Vmax*\left(\frac{Xu5P}{Km^{Xu5P}}\right)*\left(1-\frac{Ru5P}{Xu5P*K_{eq}}\right)}{\left(1+\frac{Ru5P}{Km^{Ru5P}}+\frac{Xu5P}{Km^{Xu5P}}\right)}$$

prk

$$\frac{\left(\frac{Ki^{PEP}}{PEP+Ki^{PEP}}\right)*\left(\frac{Ki^{ADP}}{ADP+Ki^{ADP}}\right)*Vmax*\left(\frac{Ru5P*ATP}{Km^{Ru5P}*Km^{ATP}}\right)*\left(1-\frac{RuBP*ADP}{Ru5P*ATP*K_{eq}}\right)}{\left(1+\frac{Ru5P}{Km^{Ru5P}}+\frac{RuBP}{Km^{RuBP}}\right)*\left(1+\frac{ATP}{Km^{ATP}}+\frac{ADP}{Km^{ADP}}\right)}$$

pgm

$$\frac{Vmax*\left(\frac{P3G}{Km^{P3G}}\right)*\left(1-\frac{P2G}{P3G*K_{eq}}\right)}{\left(1+\frac{P3G}{Km^{P3G}}+\frac{P2G}{Km^{P2G}}\right)}$$

eno

$$\frac{Vmax*\left(\frac{P2G}{Km^{P2G}}\right)*\left(1-\frac{PEP}{P2G*K_{eq}}\right)}{\left(1+\frac{P2G}{Km^{P2G}}+\frac{PEP}{Km^{PEP}}\right)}$$

pyk

$$\frac{\left(\frac{R5P}{R5P+Ka^{R5P}}\right)*\left(\frac{Ki^{P_i}}{P_i+Ki^{P_i}}\right)*\left(\frac{Ki^{ATP}}{ATP+Ki^{ATP}}\right)*\left(\frac{Ki^{ATP}}{ATP+Ki^{ATP}}\right)*Vmax*\left(\frac{PEP*ADP}{Km^{PEP}*Km^{ADP}}\right)\left(1-\frac{PYR*ATP}{PEP*ADP*K_{eq}}\right)}{\left(1+\frac{PEP}{Km^{PEP}}+\frac{PYR}{Km^{PYR}}\right)*\left(1+\frac{ATP}{Km^{ATP}}+\frac{ADP}{Km^{ADP}}\right)}$$

pdh

$$\frac{Vmax*\left(\frac{PYR*NAD*COA}{Km^{PYR}*Km^{NAD}*Km^{COA}}\right)*\left(1-\frac{ACCOA*NADH*CO_{2}\_cyt}{PYR*NAD*COA*K_{eq}}\right)}{\left(1+\frac{NAD}{Km^{NAD}}+\frac{NADH}{Km^{NADH}}\right)*\left(1+\frac{ACCOA}{Km^{ACCOA}}+\frac{COA}{Km^{COA}}\right)*\left(1+\frac{PYR}{Km^{PYR}}+\frac{CO_{2}\_cyt}{Km^{CO_{2}\_cyt}}\right)}$$

*xfpk*1

$$\frac{Vmax*\left(\frac{F6P*P_i}{Km^{F6P}*Km^{P_i}}\right)*\left(1-\frac{E4P*ACETP}{F6P*P_i*K_{eq}}\right)}{\left(1+\frac{F6P}{Km^{F6P}}+\frac{E4P}{Km^{E4P}}\right)*\left(1+\frac{P_i}{Km^{P_i}}+\frac{ACETP}{Km^{ACETP}}\right)*\left(1+\frac{Xu5P}{Km^{Xu5P}}+\frac{GAP}{Km^{GAP}}\right)}$$

*xfpk*2

$$\frac{Vmax*\left(\frac{Xu5P*P_i}{Km^{Xu5P}*Km^{P_i}}\right)*\left(1-\frac{GAP*ACETP}{Xu5P*P_i*K_{eq}}\right)}{\left(1+\frac{F6P}{Km^{F6P}}+\frac{E4P}{Km^{E4P}}\right)*\left(1+\frac{P_i}{Km^{P_i}}+\frac{ACETP}{Km^{ACETP}}\right)*\left(1+\frac{Xu5P}{Km^{Xu5P}}+\frac{GAP}{Km^{GAP}}\right)}$$

pta

$$\frac{Vmax*\left(\frac{ACETP*COA}{Km^{ACETP}*Km^{COA}}\right)*\left(1-\frac{ACCOA*P_i}{ACETP*COA*K_{eq}}\right)}{\left(1+\frac{ACETP}{Km^{ACETP}}+\frac{ACCOA}{Km^{ACCOA}}\right)*\left(1+\frac{COA}{Km^{COA}}+\frac{P_i}{Km^{P_i}}\right)}$$

ATPSyn

$$\frac{Vmax*\left(ADP*P_i-\frac{ATP}{K_{eq}}\right)}{Km^{ADP}*Km^{P_i}*\left(\left(1+\frac{ADP}{Km^{ADP}}+\frac{P_i}{Km^{P_i}}+\frac{ATP}{Km^{ATP}}\right)+\left(\frac{ADP*P_i}{Km^{ADP}*Km^{P_i}}\right)\right)}$$

NADPase

$$\frac{Vmax*\left(\frac{NADP}{Km^{NADP}}\right)*\left(1-\frac{NADPH}{NADP*K_{eq}}\right)}{\left(1+\frac{NADP}{Km^{NADP}}+\frac{NADPH}{Km^{NADPH}}\right)}$$

Sink_R5P

$$\frac{Vmax*R5P}{R5P+Km^{R5P}}$$

Sink _F6P

$$\frac{Vmax*F6P}{F6P+Km^{F6P}}$$

Sink_E4P

$$\frac{Vmax*E4P}{E4P+Km^{E4P}}$$

Sink_PEP

$$\frac{Vmax*PEP}{PEP+Km^{PEP}}$$

Sink_PYR	$\frac{Vmax*PYR}{PYR+Km^{PYR}}$
Sink_3PG	$\frac{Vmax*P3G}{P3G+Km^{P3G}}$
Supply_P_i	$K*(PPool-P_i)$

Supplementary Protocol S3. Additional information about flux balance analysis conditions and thermodynamic evaluation of sampled metabolite concentration sets.

Defining the "Metabolic State"

The size of the metabolite pools is object to the enzymatic reactions acting on this pool, with the change if these pools defined as:

$$\frac{dX}{dt} = S \cdot v(X) \quad (2)$$

Where all *m* metabolites are represented in the *m*-dimensional vector X, all *n* fluxes are represented in the *n*-dimensional vector *v*. The magnitude of the fluxes depends on the metabolite concentration according to their rate equation. S is the *m* x *n*-dimensional stoichiometric matrix and *d*X/*dt* marks the change of the metabolite concentrations over time, which must be zero at steady state:

$$\frac{dX}{dt} = S \cdot v(X^0) = 0 \quad (3)$$

with X^0 as the metabolite concentrations at steady state.

Determination of Steady State Flux Distribution via FBA

FBA on a GEM of *Synechocystis* was performed via the COBRA-toolbox (Schellenberger *et al.*, 2011) in MATLAB (v. R2015a), simulating autotrophic growth. All reversible reactions were separated into two reactions, representing the corresponding forward and backward reaction.

To obtain the flux distribution, we first set biomass production as the objective function to be maximized. Biomass production was then fixed at its optimum and all other fluxes were minimized.

The GEM was constrained by a photon influx of 50 mmol·gDCW-1·h-1 and an active HCO_3 uptake limited to a maximum of 3.7 mmol·gDCW-1·h-1. Uptake of organic carbon

sources was blocked. General metabolic constraints were employed as described in the supporting information of the original publication of the GEM (Knoop *et al.*, 2013).

The *pta* reaction, converting ACETP to ACCOA, was set to be reversible.

We set a lower limit of the *pdh* and *xfpk* fluxes to 0.4 and 0.02 mmol·gDCW-1·h-1, respectively, to ensure realistic flux through these reactions.

All FBA results had to be manually adjusted to account for small fluxes that occurred in the context of the genome-scale network and violated the steady state assumption when applied to the small-scale model. Furthermore, the flux from acetate towards ACETP was omitted. The fluxes for each sink reaction were the sums of all fluxes using this metabolite not covered by the model structure (see Fig. 1B). The fluxes for the two reactions representing the photosystems were set to values balancing the usage of the respective co-factor by other reactions in the model, as they abstracted only the fraction of the light reactions fueling the Calvin cycle.

To translate the flux distribution from the FBA output in mmol·gDCW-1·h-1 to mM·min-1, a cellular density of 434.78 gDCW·L-1 was assumed, as described for *Escherichia coli* in (Bennett *et al.*, 2009).

The final flux distributions used for the parameter sampling are listed in Table S2.

Sampling of thermodynamically feasible metabolite concentration sets

A previous study by Asplund-Samuelsson *et al.* (2018), used a large-scale metabolic model to explore the allowable metabolite concentration space in *Synechocystis* constrained by reaction directionality and equilibrium constants via Network Embedded Thermodynamic (NET) analysis (Kümmel *et al.*, 2006; Zamboni *et al*., 2008). Published metabolomics data was used to further constrain the feasible concentration space. The analysis resulted in a concentration range for each metabolite in which thermodynamics allowed the associated reactions in the network to carry flux in the direction required for biomass formation (see Table S3).

To reduce the apparent uncertainty about the metabolic state of the system, random sampling of the metabolite concentrations was performed. The resulting sets of specific metabolite concentrations were necessary to sample the kinetic parameters in the kinetic parameter sampling.

For each metabolite, a random value within the previously determined concentration ranges (Asplund-Samuelsson *et al.*, 2018) was sampled in log space, resulting in sets of metabolite concentrations spanning the concentration space evenly and unbiased for higher concentrations.

Note that the concentrations were fixed at 0.273 mM for O_2, and 0.15 mM and 0.0136 mM for carboxysomal and cytosolic CO_2, respectively (Asplund-Samuelsson *et al.*, 2018).

The resulting sets were tested for their thermodynamic feasibility by calculating the change in Gibbs Free Energy using

$$\Delta_r G = \left(ln(Q) - ln\left(K_{eq}\right)\right) \cdot R \cdot T \tag{2}$$

with equilibrium constants (K_{eq}) taken from Equilibrator (Flamholz *et al.*, 2012) at pH 8.4 (Mangan *et al.*, 2016) and ionic strength of 0.1 M. R denotes the universal gas constant , T the total temperature with T = 303.15 K and Q the quotient of product concentrations over substrate concentrations.

For the cofactor-pairs ATP/ADP, NADP/NADPH, NAD/NADH, additional constraints on their ratio were placed, respectively. Both metabolites of each pair were sampled randomly as all other metabolites, but when their ratio towards each other was outside the pre-defined range (see Supplementary Table S3 and Asplund-Samuelsson *et al.*, 2018), the sampling was repeated until the ratios were in the pre-defined range.

For the reaction abstracting the ATP supply from photosynthesis (*ATPSyn*), the K_{eq} was set to allow only the forward direction in the concentration ranges of ATP, ADP and P_i. The K_{eq} for NADPH recycling (*NADPase*) was taken from the electron-transfer reaction from ferredoxin (EC 1.18.1.2).

Concentration sets violating the thermodynamic filter by not providing every reaction with a negative change in Gibbs Free Energy in the predefined direction were discarded.

Implementation of the free phosphate supply

The drain of phosphorylated metabolites via the sink reactions required a reaction, replenishing the free inorganic phosphate P_i to fulfil the steady state conditions. Since a

detailed modelling of the rather complex phosphate metabolism in *Synechocystis* was beyond the scope of this study, the supply with P_i was implemented as a reaction representing an abstracted sum of the P_i-providing metabolism, called *Supply_P$_i$*. This reaction provided P_i from an abstracted pool of all non-free phosphate in the cell, termed PPool, and followed mass action kinetics with equal forward and reverse rate constants (see Supplementary List S1). With the rate constant K depending on the difference between the P_i- and PPool concentration, which was sampled (see **Input: Generation of feasible metabolomes using random sampling**), a wide range of possible dynamics were covered.

Accordingly, a small difference between P_i and PPool resulted in a large K and fast dynamics, whereas a large difference between P_i and PPool resulted in a small K, resembling slow dynamics. By sampling in between these two extremes, many different dynamic scenarios for the phosphate supply were covered.

References

Asplund-Samuelsson J, Janasch M, Hudson, EP. 2018. Thermodynamic analysis of computed pathways integrated into the metabolic networks of E. coli and Synechocystis reveals contrasting expansion potential. Metabolic Engineering **45**, 223–236.

Bennett B, Kimball E, Gao M, Osterhout R, Van Dien SJ, Rabinowitz JD. 2009. Absolute metabolite concentrations and implied enzyme active site occupancy in Escherichia coli. Nature Chemical Biology **5**, 593–599.

Flamholz A, Noor E, Bar-Even A, Milo R. 2012. eQuilibrator – the biochemical thermodynamics calculator. Nucleic Acids Research **40**, 770–775.

Knoop H, Gründel M, Zilliges Y, Lehmann R, Hoffmann S, Lockau Wolfgang, Steuer R. 2013. Flux Balance Analysis of Cyanobacterial Metabolism: The Metabolic Network of Synechocystis sp. PCC 6803. PLoS Computational Biology **9**, e1003081.

Kümmel A, Panke S, Heinemann M. 2006. Putative regulatory sites unraveled by network-embedded thermodynamic analysis of metabolome data. Molecular Systems Biology **2**, doi:10.1038/msb4100074.

Mangan NM, Flamholz A, Hood RD, Milo R, Savage DF. 2016. pH determines the energetic efficiency of the cyanobacterial CO_2 concentrating mechanism. Proceedings of the National Academy of Sciences **113**, E5354-E5362.

Schellenberger J, Que R, Fleming RMT, *et al*. 2011. Quantitative prediction of cellular metabolism with constraint-based models: the COBRA Toolbox v2.0. Nature Protocols **6**, 1290-1307.

Zamboni N, Kümmel A, Heinemann M. 2008. anNET: a tool for network-embedded thermodynamic analysis of quantitative metabolome data. BMC bioinformatics **9**, 199.

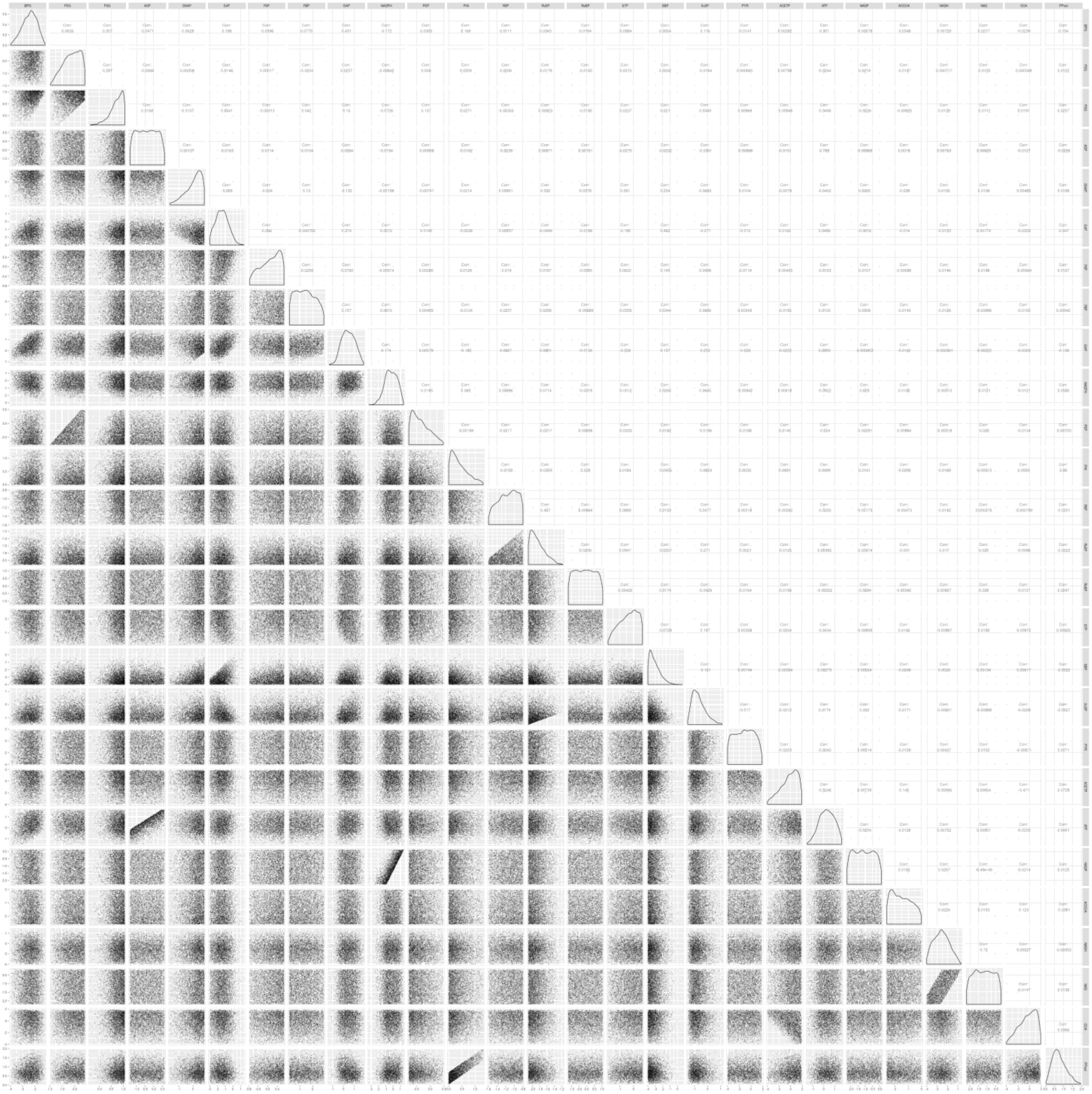

Fig. S1. Distribution of sampled metabolite concentrations and correlations between the metabolite concentrations for all feasible metabolite concentration sets. All axes in log10-scale.

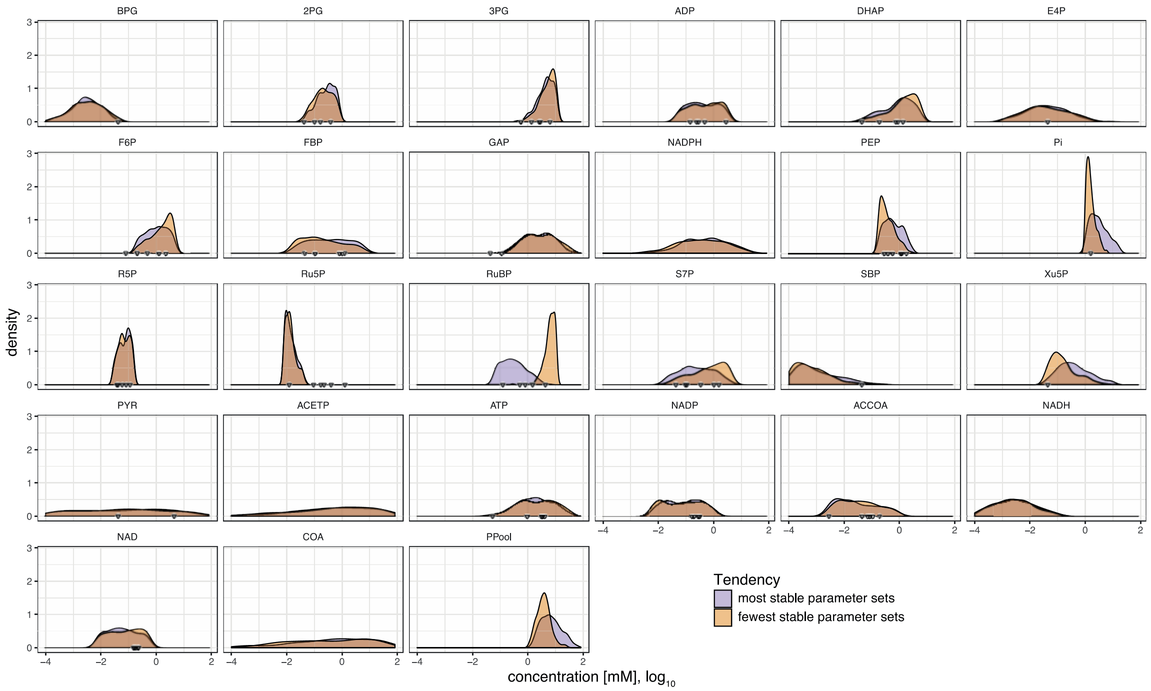

Fig. S2. Tendencies of all sampled metabolite concentrations towards stability. Density of metabolite concentrations in the top (purple; most stable parameter sets) and bottom (orange; fewest stable parameter sets) deciles of fMCSs according to number of stable steady states. Concentration is depicted in a log10 scale on the X-axis. Triangles on the X-axis indicate published values used for determining concentration ranges in Asplund-Samuelsson *et al.* (2018).

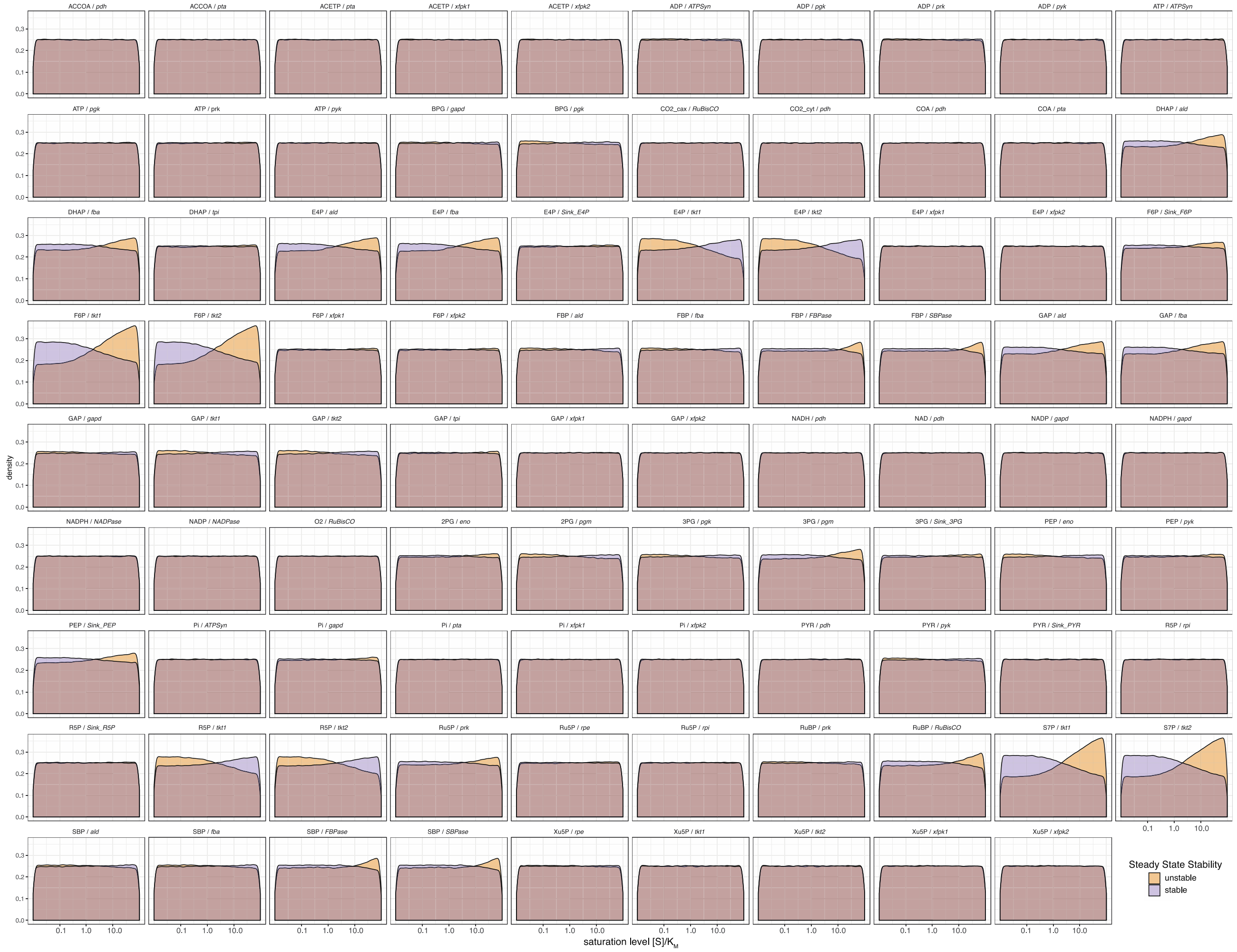

Fig. S3. Tendencies of enzyme saturation states towards causing stable and unstable systems. Density of all enzyme saturation levels, defined as the metabolite concentration divided by the associated K_m-value, depicted on a log10 scale. Purple and orange refer to stable and instable states, respectively. The header for each histogram states the metabolite-reaction pair the saturation refers to.

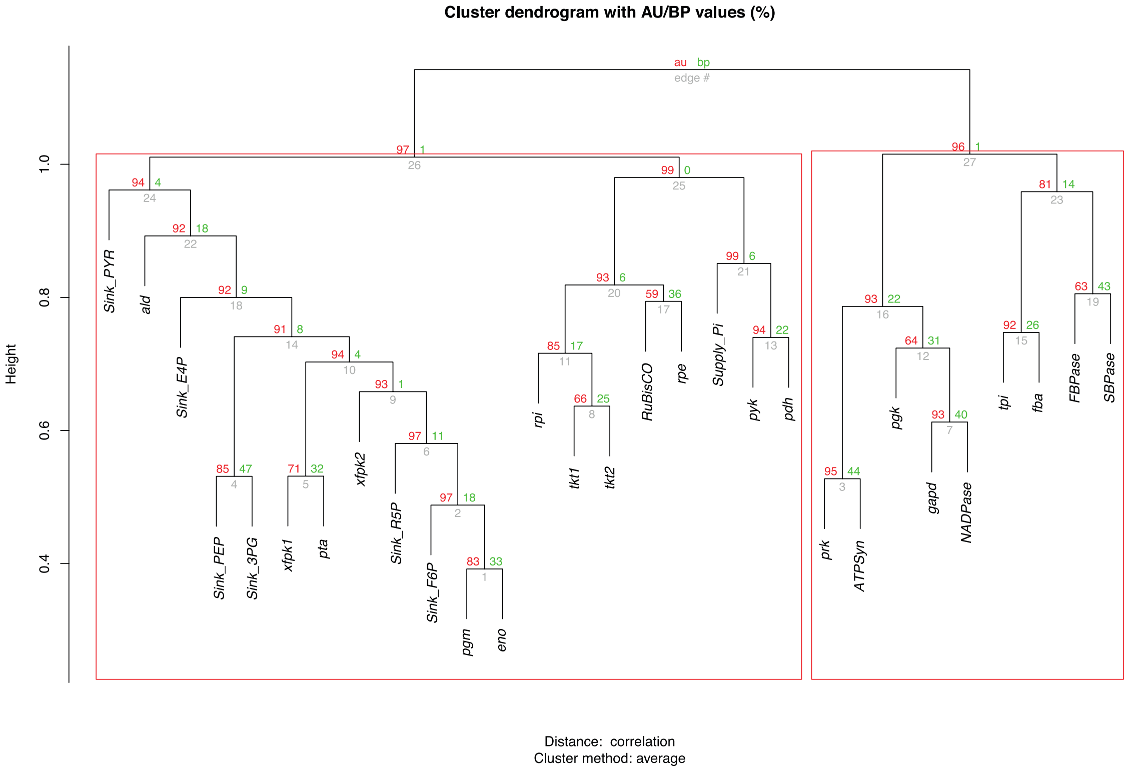

Fig. S4. Clustering of reactions according to their property of exerting control, *i.e.* effectors in Fig. 4. Effector-clustering of FCC pattern was carried out as described the Models and Methods. Approximately unbiased p-values (AU) above 95 indicate significantly similar FCC pattern.

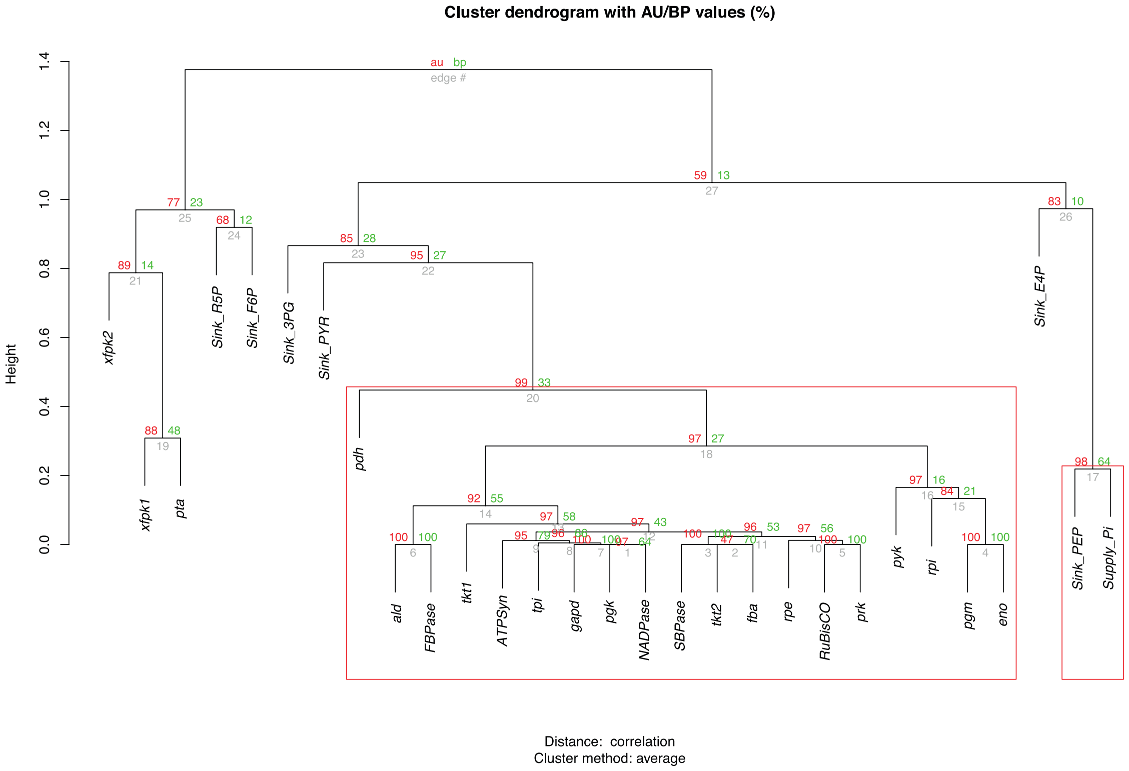

Fig. S5. Clustering of reactions according to their property of being controlled, *i.e.* targets in Fig. 4. Target-Clustering of FCC pattern was carried out as described the Models and Methods. Approximately unbiased p-values (AU) above 95 indicate significantly similar FCC pattern.
